# What Are the Determinants of the Decision to Purchase Private Health Insurance in China?

**DOI:** 10.3390/ijerph17155348

**Published:** 2020-07-24

**Authors:** Guangsheng Wan, Zixuan Peng, Yufeng Shi, Peter C. Coyte

**Affiliations:** 1 School of Nursing and Health Management, Shanghai University of Medicine & Health Sciences, 279 Zhouzhu Highway, Pudong New Area, Shanghai 201318, China; shiyf@sumhs.edu.cn; 2 Institute of Health Policy, Management and Evaluation, University of Toronto, Health Sciences Building, 155 College Street, Toronto, ON M5T3M6, Canada; zixuan.peng@mail.utoronto.ca (Z.P.); peter.coyte@utoronto.ca (P.C.C.)

**Keywords:** China, private health insurance, determinants of purchasing decision, socio-economic status, health system

## Abstract

The objective of this study was to assess the determinants of the decision to purchase private health insurance (PHI) in China. Nationally representative data from the fourth wave of the China Household Finance Survey from 2017 were used, and the dataset comprised 105,691 individuals aged 18 years or older. The Andersen health services utilization model was used to inform the research. Chi-square tests and logistic regression analyses were used to estimate the decision to purchase PHI. The proportion of the sample that had PHI was small, at 5.06%, but coverage for social basic medical insurance (SBMI) was 90.64%. Among PHI holders, the overwhelming majority (87.40%) also had SBMI. Logistic regression analysis demonstrated that predisposing factors (age, education, marital status, household size), enabling factors (household income, SBMI status, geographical factors, household medical expense, and medical debt), and needs-based factors (health status) were statistically significant determinants of the decision to purchase PHI. This study suggests that the socio-economic circumstances of households play a crucial role in the decision to acquire PHI. The findings may be used by the insurance industry to inform actions to enhance PHI coverage and by policy decision-makers that seek to improve equality in access to PHI.

## 1. Introduction

For more than three decades, the Chinese government has devoted considerable efforts to expanding universal health insurance coverage [1]. Three main programs provide insurance coverage for various groups in Chinese society. Firstly, the Urban Employee Basic Medical Insurance (UEBMI) scheme was piloted in 1994 and was launched nationwide in 1998. This scheme provides coverage to employees in formal sectors and is funded by both employees and their employers. Secondly, the New Cooperative Medical Scheme (NCMS) was set up in 2003 and provides coverage for rural residents. Households and central and local governments fund it. Thirdly, the Urban Resident Basic Medical Insurance (URBMI) scheme was piloted in 2007 and then implemented nationwide in 2009. It was designed to provide coverage to urban residents who were not covered by the UEBMI scheme [2]. Both URBMI and NCMS were funded jointly by those insured and by the government [3], while UEBMI was funded by those insured and their employers. Due to the disparities caused by the market segmentation of health insurance (UEBMI, NCMS, and URBMI) in China, different sections of the population face different degrees of financial protection [4,5]. Since 2016, the Chinese government has moved forward with reforms to the existing schemes. The first step was taken in 2016 to integrate URBMI and NCMS and to reshape the Urban and Rural Resident Basic Medical Insurance (URRBMI) scheme. The URRBMI scheme unified protection standards for urban and rural residents. Therefore, UEBMI, NCMS, URBMI, and URRBMI co-existed in 2017. Besides these, the Free Medical Service (FMS), which provides free medical services for staff in government or public institutions, was set up in 1952 and has been gradually phased out since 1998. The above schemes provide basic healthcare protection for various groups and are all mandatory health insurance schemes. As such, these schemes are collectively named Social Basic Medical Insurance (SBMI) in Chinese society. Coverage under all forms of SBMI reached 95% in 2011 [6,7]. According to the *Statistical Bulletin on the Development of Medical Security in 2019*, announced by the China National Healthcare Security Administration on 30 March 2020, the number of people covered by SBMI was 1354.36 million. Once combined with population data from the China National Bureau of Statistics, we determined that 96.7% of Chinese residents were covered by the SBMI.

Private health insurance (PHI) was initially introduced in China in the 1980s [8], and it helped to expand the breadth of universal health coverage (UHC) in the absence of SBMI. With the rapid increase in income in the last 20 years, individuals now seek enhancements to the quality of healthcare [9]. However, SBMI is limited in service coverage and reimbursement. It is difficult for SBMI to meet enrollees’ growing needs for higher quality medical services [10]. While PHI can provide a broader range of coverage options [5], the central government has begun to attach importance to developing PHI since 2014. The State Council of China announced *Some Opinions on Accelerating the Development of Commercial Health Insurance* in November 2014 [11]. This policy encouraged commercial insurance companies to run PHI businesses. It expected that PHI and SBMI could promote each other and improve the multi-tier medical security system. The *Health China 2030 Strategy* was announced in May 2016, with encouragement of the development of PHI as one central platform. On 1 July 2017, preferential tax policies for PHI were officially implemented to stimulate the development of PHI. In China’s system, the SBMI mandates minimum insurance coverage and allows for purchasing additional voluntary PHI [12].

More attention should be paid to the PHI market and purchasing behavior in China. Systematic research on health insurance has shown that more attention was paid to SBMI than to PHI—for example, the impact of the NCMS on the reduction of out-of-pocket medical expenditures, thereby improving financial protection [13,14,15], and the role played by other schemes such as UEBMI in containing health expenditures [16]; the role of SBMI in lowering catastrophic health expenditures and increasing outpatient expenditure reimbursement [1,2,3,17,18]. Studies on PHI have mainly focused on the way in which the expansion of SBMI has affected the development of PHI [19]. Studies in other countries indicated that these factors, such as an individual’s risk preference/aversion, cognitive ability, expected out-of-pocket cost, and personal characteristics (age, education, income, health status), determined the selection of PHI [20,21,22]. Determinants of PHI coverage have not been fully discussed in the context of China, and most studies have discussed PHI in very general terms [23]. It is now opportune to assess the development of PHI more completely. Indeed, it is crucial to study the determinants of the decision to purchase PHI in China. In this study, we use the Anderson model of health service utilization as the key conceptual framework in understanding the determinants of PHI purchasing behavior.

The purpose of this study is to assess the determinants of the decision to purchase PHI. The next section briefly discusses the conceptual framework. The Section 3 discusses the data and methods, and Section 4 outlines the study results. We discuss our findings in the context of the literature in Section 5, and we conclude with a brief set of policy implications.

## 2. Conceptual Framework

The Andersen model of health service utilization provides the underlying conceptual basis for our empirical analysis. This conceptual framework has been used in the literature to account for variations in health services utilization [24,25,26]. The model advances three sets of factors to account for health services utilization and, in this paper, the determinants of the decision to purchase private health insurance. These three factors are predisposing, enabling, and needs-based factors [27,28]. Each of these determining factors are described below.

Predisposing factors: these represent factors that make it more likely that individuals will seek health services. These include demographic variables, such as age and gender, which influence a person’s likelihood of purchasing PHI. Additionally, social variables, such as marital status, education level, and household size, are variables that predispose individuals to acquire PHI.

Enabling factors: these represent factors that support health-seeking behavior and, in our context, provide support for individuals to purchase PHI. These include household income, as the main unit of decision-making in Chinese society is the household unit, and variables that represent employment status, as each type of employment circumstance offers differential access to other types of health insurance that may either substitute for or complement the decision to acquire PHI. Moreover, place of residence and household registration status (i.e., Hukou status) provide individuals with different opportunities to access services and thereby influence their decision to purchase PHI. Household medical expense is also considered, as these expenses capture the scale of financial protection sought by individuals in their decision to purchase PHI. We also included medical debt status as a variable to support their seeking behavior.

Needs-based factors: these represent factors associated with health status that generate an underlying need to acquire health services and consequently result in a derived demand for PHI. These variables include perceived and evaluated health status, as these variables are prerequisites for health service utilization.

## 3. Data and Methods

### 3.1. Data Source

Data were obtained from the fourth wave of the China Household Finance Survey (CHFS) in 2017. This survey was launched in 2009 by the Survey and Research Center for China Household Finance, which conducts nationwide surveys every two years. A three-stage stratified random sampling design method was used in the survey. First, counties were randomly selected based on GDP groups (ranked according to GDP and classified into groups) from 2585 counties in all provincial administrative regions excluding Xinjiang, Tibet, Macao, Hong Kong, and Taiwan. Second, within each country selected, there was random sampling of communities and then the random selection of 25–50 households within each urban community and 20 households for rural communities [29,30]. Finally, face-to-face interviews were conducted with adults in selected households. Details of the design of CHFS can be found on their website: https://chfs.swufe.edu.cn/science/family.html.

The CHFS represents the most comprehensive public source for household finances in China. As the decision to purchase PHI is strongly influenced by household financial status, the growth in household finance associated with the dramatic growth of the Chinese economy has resulted in a burgeoning of PHI. Over the past five years, with the steady growth in PHI, more attention has been paid to this sector by the central Chinese government. We use, in our analysis, the most recently available public data to identify the determinants of the decision to purchase PHI. The fourth survey used in our analysis covered 29 provinces, 355 counties, and 1428 villages, with a sample size of 40,011 households that in total comprised 127,012 individuals. Our research focused on individuals aged 18 years or older who were asked to complete information on their decision to purchase PHI. This resulted in an analysis sample of 105,691 individuals, with 99.54% reporting PHI purchasing information. 

### 3.2. Statistical Analysis

The dependent variable was binary and indicated whether an individual bought PHI or did not purchase such insurance. The independent variables were summarized previously under the three sets of factors suggested by the Anderson model, namely predisposing, enabling, and needs-based factors, and these variables are listed in Table 1.

Logistic regression analysis and chi-square tests were used to assess the determinants of the decision to purchase PHI empirically. First, descriptive statistics and chi-square tests of independence were calculated in order to assess the whole data set (Table 2 and Table 3). Second, univariate logistic regression analysis was applied to examine the crude relationship between each independent variable and the decision to purchase PHI (Table 4, Model 1). All the results of model fit from univariate logistic regression are reported in Appendix A. Third, multivariable logistic regression analysis was performed based on the results of the univariate analysis. Backward stepwise logistic regression analysis was performed with a significance level of 0.10 to enter variables into the multivariate model (Table 4, Model 2). The results of Model 2 were statistically significant ($\chi^{2}$ = 5783.55, *p* = 0.000), indicating that the model was able to distinguish between respondents who purchased PHI and those who did not purchase PHI. Other models were also performed to assess model robustness (Appendix A Table A1).

All of the logistic regression results were reported as odds ratios (ORs) and 95% confidence intervals (CIs). An OR above one indicates that the specified determinant was more likely to be associated with PHI purchasing. STATA 15.0 was used to perform the data analysis.

## 4. Results

### 4.1. Characteristics of the Sample

Table 2 reports the descriptive statistics for the study variables. Of the 105,691 respondents, 5344 (or 5.06%) reported that they had purchased PHI. The sample was almost equally divided between men and women (49.77% vs. 50.23%). The proportion of individuals in each age group was relatively balanced, and the largest proportion was in the group aged 65 or over (21.61%). More than 79% of the respondents were married. Around 65.18% of the sample were from urban regions, and 48.73% were from the eastern area of China. Chi-square tests of independence were used to analyze the difference between the characteristics of those that purchased PHI and those that did not make such purchases. Table 2 reports the preliminary analysis of chi-square tests of independence and demonstrates, as expected, that there were significant differences in all variables (except the continuous independent variable, household size).

### 4.2. PHI Purchasing and SBMI Holding

Table 3 reports the descriptive information on respondents with both PHI and SBMI. The overwhelming majority (90.64%) of the total sample had one of the four primary forms of SBMI. The overwhelming majority (87.4%) of individuals who purchased PHI also had some form of SBMI. For those with both forms of insurance, almost half (46.6%) also had UEBMI.

### 4.3. Logistic Regression Model

Table 4 reports the results of the logistic regression analysis. The univariate logistic regression suggested that all the potential factors were significantly associated with PHI purchasing (Model 1, Table 4). Men, married respondents, urban residents, and those living in the eastern area of China tended to report a higher likelihood of purchasing PHI than the reference group. Education level and household income were also positively related to this likelihood. Household size and medical debt were negatively related to the likelihood.

The multivariable logistic regression analysis was performed by using backward stepwise regression methods (Model 2, Table 4). This analysis identified the variables that were statistically significant determinants of the decision to purchase PHI. We also checked the collinearity of the independent variables. The variance inflation factor (VIF) for all the independent variables in Model 2 ranged from 1.02 to 4.08, with all values below the conventional threshold value, and the maximum VIF was below 10 [31]. This result indicated no serious collinearity problems. We applied the Anderson model as the conceptual framework to guide our empirical work and listed independent variables under each of the three sets of factors, which were expected to be associated with the decision to purchase PHI. The findings are discussed in line with each of these factors.

In the case of predisposing factors, namely age, education, marital status, and household size, each was seen to be a significant independent variable in driving decisions to purchase PHI. Individuals with higher levels of educational achievement exhibited higher odds of purchasing PHI. Compared with the reference group, individuals aged 25–54 years old were more likely to buy PHI. Being married increased the likelihood that PHI would be purchased by 18.0% (OR, 1.180; 95% CI, 1.047–1.330). While marital status was significantly related to the decision to purchase PHI, household size was negatively associated with the same decision. The results demonstrated that a unit increase in the number of household members was associated with a reduction in the odds of purchasing PHI by 10.9% (OR, 0.891; 95% CI, 0.866–0.916).

For enabling variables, we were not able to find a statistically significant role played by either Hukou type or employment status in the decision to purchase PHI. In contrast, we did find a critical role placed by geographic factors. Specifically, residents from eastern regions had a higher propensity to acquire PHI than those in the middle regions of China, with the lowest propensity to purchase PHI occurring in western regions. Residents in western regions were 18.2% less likely to acquire PHI than those in eastern regions (OR, 0.818; 95% CI, 0.743–0.901). Rural residents were 11.0% less likely to buy PHI than urban residents (OR, 0.890; 95% CI, 0.797–0.994). For those who were currently working, the type of work unit influenced the odds of purchasing PHI. Employees of state-owned/collective enterprises and private/foreign-owned enterprises were more likely to acquire PHI than those in other types of enterprises. Income was an essential factor in the purchasing decision. The results showed that the likelihood of purchasing PHI increased with household income. SBMI is a mandatory form of health insurance in China, but there are limits to its medical service coverage and the level of reimbursement. When the government liberalized the commercial health insurance market, there was a growth in the decision to purchase PHI in order to obtain more and better coverage, even when individuals were still holding SBMI. The likelihood of purchasing PHI varied by the type of SBMI that individuals held. Individuals were 16.1% less likely to acquire PHI, relative to those covered by the UEBMI scheme, if they were covered by the NCMS (OR, 0.839; 95% CI, 0.745–0.994), but they were 36.1% more likely to purchase PHI if they were covered by URRBMI (OR, 1.361; 95% CI, 1.092–1.698). Individuals without any form of SBMI were 67.1% more likely to purchase PHI (OR, 1.670; 95% CI, 1.458–1.913). Moreover, because the decision to purchase insurance is correlated with financial risk perception, our expectations were confirmed when the decision to purchase PHI was significantly associated with decisions by individuals who had bought other types of private non-health insurance. Those that made such purchases were much more likely—indeed, nine-fold more likely—to buy PHI than those that had not made such decisions (OR, 10.222; 95% CI, 9.395–11.122). It was also shown that an increase in medical expenditure increased the likelihood to purchase PHI. Specifically, when household annual medical spending was in the 2000–4999 RMB and 5000–9999 RMB groups, the decision to purchase PHI was 12.9% and 16.4% more likely, respectively, than for those in the 0–1999 RMB group (OR, 1.129; 95% CI, 1.027–1.242 and OR, 1.164; 95% CI, 1.037–1.306). In more extreme expenditure situations—i.e., where expenditure exceeded 10,000 RMB—there was no statistically significant relationship to report, as such levels of expenditure might drive the household into debt. Indeed, where respondents reported medical debt, the likelihood that they would acquire PHI was significantly reduced, by 29.2%, compared to those without such debts (OR, 0.718; 95% CI, 0.548–0.942).

Needs-based factors were also shown to be significant determinants of the decision to purchase PHI. Individuals with poor self-evaluated health were less likely to buy PHI than individuals reporting good or fair health. Existing studies indicated that healthier individuals had higher risk aversion, and purchasing PHI was more suggestive of risk aversion [21]. This might be the reason that healthier individuals were more likely to buy voluntary PHI.

We also conducted other multivariable logistic regression models in order to acquire robust results. First, a multivariable model with all the potential predictors included was performed, and the results are reported in Appendix A (Table A1, Model 4). Second, considering potential bias caused by data where employer type was missing, the multivariable model with all the potential predictors except employer type was performed (Table A1, Model 3). The main results were consistent with our results above. We also performed models with each set of factors individually to show the model improvements when considering all three sets of factors (Table A2, Model 5–7).

## 5. Discussion

This study represents the first examination of the determinants of the decision to purchase PHI in the context of China. We found that 5.06% of the study sample had purchased PHI, and 87.4% of those with PHI also had other forms of insurance coverage through SBMI. The results demonstrate the usefulness of the Anderson model, which highlights the role of predisposing factors (age, education, marital status, household size), enabling factors (household income, SBMI status, geographical factors, household medical expense, and medical debt), and needs-based factors (health status) as statistically significant determinants of the decision to purchase PHI.

Health and household financial status were significant determinants of the decision to buy PHI. Individuals with higher health status were more likely to acquire PHI. This finding is consistent with US studies that have shown that those with better health status are more likely to buy private Medigap insurance [32,33]. Higher socioeconomic status was also correlated with the acquisition of PHI [34]. Household financial status affects the decision to purchase health insurance, which in turn might impact out-of-pocket household health expenditure. For example, several studies have discussed catastrophic health expenditure (CHE), because CHE directly leads to medical debt and there is still a high percentage of CHE in Chinese households [35]. It was found that NCMS increased out-of-pocket medical expenditure by 12.3% [14], and it failed to prevent CHE and medical impoverishment [15]. In response to this situation, the Chinese government introduced, in 2016, the critical illness insurance (CII) program, which provides coverage to alleviate economic burdens among those with NCMS and URBMI coverage [36]. However, it is still unknown whether this program has achieved its intent [35]. To the best of our knowledge, few studies have addressed the effects of PHI on CHE in the context of China.

It is hard to say that PHI will provide financial protection for most of the people in China because of the complicated relationship between PHI and SBMI. PHI was expected to expand universal healthcare insurance coverage and alleviate disease burden, but there has been significant controversy regarding the relationship between PHI and SBMI. As for how SBMI affected PHI, Zhang et al. (2018) pointed out that the impacts of SBMI on PHI were different in terms of insurance penetration and density [11]. Studies in the context of other countries revealed that there was a negative (or crowding-out) relationship. For example, evidence from the US using Medicaid data indicated that the expansion of public health insurance was associated with lower private market insurance premiums [37]. Our study indicated that different types of SBMI might impact the demand for PHI in the context of China. However, we must pay attention to the differences in the health system context between China and other countries. More than 95% of the residents of China are enrolled in the SBMI, which is a mandatory form of insurance coverage, and only a small portion of those individuals also voluntarily acquired PHI. Among SBMI holders (including UEBMI, NCMS, URBMI, URRBMI, and FMS), 4.83% of them purchased PHI. The main point is that the health insurance system is different from the dual insurance system in countries such as Germany and Chile, where individuals only acquire either PHI or social health insurance [38]. In China, government encouragement of the expansion of PHI is not for the purpose of covering individuals who are unable to acquire SBMI. Rather, PHI in China is a form of supplementary coverage for individuals who are already enrolled in the SBMI scheme [8]. Our study revealed that PHI purchasing was significantly associated with household financial status, including household income and medical spending. As a result, the acquisition of PHI did not provide financial protection for those in financial need [23] but rather may best be seen as a mechanism to increase access to, and probably perceived quality of, health services. Consequently, it is limited in alleviating CHE and decreasing impoverishment in China.

Moreover, PHI has the potential to exacerbate health inequalities in China. Health insurance schemes were examined to have an impact on healthcare utilization and costs [39], and different health insurance schemes may be associated with healthcare utilization inequality [40]. There are demands in China to distribute access to healthcare services more equitably, in line with the population’s needs rather than their ability to pay [41]. Our findings demonstrate the importance of household financial status, geographical factors, and SBMI type as associated with the decision to purchase PHI. Different SBMI schemes caused variation in inequalities in accessing healthcare [40], and the acquisition of PHI might further exacerbate this inequality. This warrants study in future work.

The results of this study demonstrated that household financial status was the critical determinant of the decision to purchase PHI. Our study has two contributions. Firstly, it can contribute to the existing literature by providing new findings about the determinants of PHI purchasing in a different relationship context between private and public health insurance. Different from PHI in Germany and other developed countries, where PHI is a substitute for public health insurance, PHI in China is supplementary of public health insurance. Secondly, the results of this study provide a reference for government to adjust health policies. The results indicate that there are signs of increasing health inequities. Due to the complicated relationship between PHI and SBMI in China, PHI might not play the role that it was expected to play. Government and policymakers need to pay more attention to this and make sure that the expansion in PHI coverage is consistent with national health policy goals.

There are several limitations associated with this study. Firstly, we used cross-sectional data in order to identify an association with the decision to acquire PHI. The PHI market is still at an embryonic stage of development and is evolving rapidly in China. The overall level of population coverage is quite small but growing. There are very few databases that offer information on the decision to acquire PHI and so our choice of data was limited to the China Household Finance Survey (CHFS). Secondly, some critical factors might have been excluded from our analysis because they were absent from the CHFS, especially data on some needs-based factors, such as physician visits, comorbid conditions, and disease related spending. Finally, the decision to purchase PHI was considered as a binary variable in our research; we did not examine the amount or form of coverage. Such details and complexities would be interesting areas of future research and also potentially open to explanation using the Anderson model of health service utilization.

## 6. Conclusions

This study contributes to our understanding of the determinants of the decision to purchase PHI in China. The socio-economic circumstances of households play a vital role in the decision to acquire PHI. Notably, household financial variables are critical determinants of the decision to acquire PHI. Moreover, it is important to monitor this area of the health services marketplace as the growth in PHI may have implications for health inequities in China. It might also be useful to both policy makers and insurance industry leaders to better understand the factors that influence decisions to acquire insurance as well as the associated depth of coverage. Policy makers may wish to monitor developments to ensure that growth in PHI is compatible with national health policy goals.

## Figures and Tables

**Table 1:** Independent variables used in the logistic regression analysis.

Predisposing Factors		
	Age	18–24 (reference group); 25–34; 35–44; 45–54; 55–64; ≥65
	Gender	Male (reference group); female
	Education	Junior high school degree and below (reference group); High school or secondary; university or college; Master’s degree or above
	Marital status	Unmarried (reference group); married
	Household size	Number of members in household (continuous)
Enabling Factors		
	Household income	<50,000 RMB (reference group); 50,000–99,999 RMB; 100,000–149,999 RMB; 150,000–199,999 RMB; ≥200,000 RMB
	Employment status	Not currently working (reference group); currently working; retired
	Employer type	Government or public institution (reference group); State-owned or collective enterprise; private or foreign-owned enterprise; land contracting operator; other
	“Hukou” type	Agricultural (reference group); non-agricultural; unity resident Hukou ^a^
	Social basic medical insurance status	UEBMI (reference group); URBMI; NCMS; URRBMI; FMS ^b^; non-SBMI
	Other private insurance	No (reference group); yes
	Geographic region	East (reference group); Central; West
	Living area	Urban (reference group); rural
	Household medical debt	No (reference group); yes
	Household medical expenses last year ^c^	<2000 RMB (reference group); 2000–4999 RMB; 5000–9999 RMB; 10,000–19,999 RMB; ≥20,000 RMB
Needs-Based Factors		
	Health status evaluation	Good (reference group); fair; poor

^a^ Due to the reforming of Hukou system, some regions of China no longer distinguish agricultural from non-agricultural Hukou. They have unified them as “unity resident Hukou”. ^b^ FMS stands for Free Medical Service; it is a kind of medical security regime that provides free or nearly free medical services for the staff in government or public institutions. It was set up in 1952 and reformed in 1998. While it has been gradually phased out since 1998, there were still a small number of employees with FMS in 2017. ^c^ Household medical expenses refer to the total household medical spending, including the insurance claim amounts and the out-of-pocket payment.

**Table 2:** Descriptive information on private health insurance (PHI) purchasing or non-purchasing under Andersen model.

Factors	Non-Purchase PHI *n* (%) (*n* = 99,866)	Purchase PHI *n* (%) (*n* = 5344)	Total Observations (*n*) ^a^ or Missing Rate ^b^ (%)	$\chi^{2}\;(\mathrm{or}\;\mathrm{Mean})$	*p* (or SD)
Gender			0.0	4.795	0.029
Male	49,610 (94.8)	2737 (5.2)	52,347		
Female	50,251 (95.1)	2607 (4.9)	52,858		
Age			0.1	1.5 × 10^3^	0.000
18–24	8476 (94.8)	469 (5.2)	8945		
25–34	14,692 (92.9)	1113 (7.1)	15,805		
35–44	14,582 (90.8)	1475 (9.2)	16,057		
45–54	20,763 (94.0)	1327 (6.0)	22,090		
55–64	18,800 (96.6)	664 (3.4)	19,464		
≥65	22,514 (98.7)	294 (1.3)	22,808		
Education			0.3	2.5 × 10^3^	0.000
Junior high school and below	63,962 (97.2)	1841 (2.8)	65,803		
High school or secondary	18,549 (93.5)	1300 (6.5)	19,849		
University or college	16,155 (89.0)	1997 (11.0)	18,152		
Master degree or above	911 (82.3)	196 (17.7)	1107		
Marital status			0.1	16.021	0.000
Unmarried	20,983 (95.4)	1001 (4.6)	21,984		
Married	78,840 (94.8)	4342 (5.2)	83,182		
Household size			0.1	3.768	1.699
Household income			0.0	2.9 × 10^3^	0.000
<50 thousand	44,393 (97.7)	1049 (2.3)	45,442		
50–100 thousand	28,184 (95.5)	1335 (4.5)	29,519		
100–150 thousand	12,977 (93.3)	935 (6.7)	13,912		
150–200 thousand	5983 (91.2)	557 (8.8)	6560		
≥200 thousand	8329 (85.2)	1448 (14.8)	9777		
Employment status			0.1	718.897	0.000
Not currently working	18,767 (94.9)	1009 (5.1)	19,776		
Currently working	55,694 (93.6)	3809 (6.4)	59,458		
Retired	25,433 (97.9)	526 (2.1)	25,959		
Employer type			42.4	955.448	0.000
Government or public institution	5642 (91.1)	548 (8.9)	6190		
State-owned or collective enterprise	5336 (88.8)	673 (11.2)	6009		
Private or foreign-owned enterprise	26,262 (92.1)	2239 (7.8)	28,501		
Land contracting operator	14,796 (98.2)	263 (1.8)	15,059		
Other	4538 (95.6)	209 (4.4)	4747		
Hukou			0.3	906.249	0.000
Agricultural	57,472 (97.7)	1956 (3.3)	59,428		
Non-agricultural	32,631 (92.6)	2608 (7.4)	35,239		
Unity resident Hukou	9461 (92.6)	760 (7.4)	10,221		
Social Basic Medical Insurance status			1.5	1.4 × 10^3^	0.000
UEBMI ^c^	23,658 (91.7)	2153 (8.3)	25,811		
URBMI ^d^	11,982 (93.7)	810 (6.3)	12,792		
NCMS ^e^	51,468 (97.3)	1412 (2.7)	52,880		
URRBMI ^f^	2530 (93.7)	171 (6.3)	2701		
FMS	1335 (94.7)	74 (5.3)	1409		
Non-SBMI ^g^	7624 (92.0)	666 (8.0)	8290		
Other private insurance			0.0	1.1 × 10^4^	0.000
No	96,477 (96.6)	3442 (3.4)	99,919		
Yes	3389 (64.1)	1902 (35.9)	5291		
Geographic region			0.0	302.897	0.000
East	48,037 (93.1)	3223 (6.3)	51,260		
Central	26,779 (96.0)	1109 (4.0)	27,888		
West	25,050 (96.1)	1012 (3.9)	26,062		
Living area			0.0	858.107	0.000
Urban	64,160 (93.5)	4480 (6.5)	68,640		
Rural	35,706 (97.6)	864 (2.4)	36,570		
Household medical debt			0.1	153.299	0.000
No	94,012 (94.7)	5248 (5.3)	99,260		
Yes	5748 (98.4)	95 (1.6)	5483		
Household medical expenses			1.9	19.163	0.001
<2000	44,502 (94.8)	2452 (5.2)	46,954		
2000–4999	18,613 (94.5)	1072 (5 5)	19,685		
5000–9999	12,409 (94.9)	665 (5.1)	13,074		
10,000–19,999	10,378 (95.4)	497 (4.6)	10,875		
≥20,000	12,150 (95.4)	586 (4.6)	12,736		
Health status			0.1	697.024	0.000
Good	50,838 (93.4)	3589 (6.6)	54,427		
Fair	30,736 (95.6)	1415 (4.4)	32,151		
Poor	18,254 (98.2)	340 (1.8)	18,594		

^a^ While the total number of respondents was 105,691, the number of observations for each variable may be less as a result of missing responses by some respondents. ^b^ Missing rate refers to the rate of missing data for each variable. The total sample size was 105,691. All the analysis was performed after eliminating missing data. ^c^ UEBMI stands for Urban Employee Basic Medical Insurance. ^d^ URBMI stands for Urban Resident Basic Medical Insurance. ^e^ NCMS stands for the New Cooperative Medical Scheme. ^f^ URRBMI stands for Urban and Rural Resident Basic Medical Insurance. ^g^ Non-SBMI stands for an individual did not hold any kind of the social basic medical insurance.

**Table 3:** Information on individuals with both PHI and SBMI in different age groups.

	SBMI Type/*n* (%)					Total (*n*)
	UEBMI ^a^	URBMI ^b^	NCMS ^c^	URRBMI ^d^	FMS ^e^	
PHI	2153 (46.6)	810 (17.5)	1412 (30.6)	171 (3.7)	74 (1.6)	4620
Age						
18–24	73 (21.8)	133 (39.7)	113 (33.7)	14 (4.2)	2 (0.6)	335
25–34	486 (51.8)	134 (14.3)	271 (28.9)	35 (3.7)	13 (1.3)	939
35–44	654 (50.2)	214 (16.4)	362 (27.8)	54 (4.1)	20 (1.5)	1304
45–54	519 (43.4)	207 (17.4)	412 (34.5)	39 (3.3)	17 (1.4)	1194
55–64	312 (51.9)	80 (13.3)	170 (28.3)	23 (3.8)	16 (2.7)	601
≥65	108 (44.1)	42 (17.1)	84 (34.3)	5 (2.0)	6 (2.5)	245

^a^ UEBMI stands for Urban Employee Basic Medical Insurance. ^b^ URBMI stands for Urban Resident Basic Medical Insurance. ^c^ NCMS stands for the New Cooperative Medical Scheme. ^d^ URRBMI stands for Urban and Rural Resident Basic Medical Insurance; this was integrated by URBMI and NCMS in 2016 and was first piloted in some provinces. ^e^ FMS stands for Free Medical Service; this is a kind of medical security regime that provides free or nearly free medical services for the staff in government or public institutions. It was set up in 1952 and reformed in 1998. While it has been gradually phased out since 1998, there were still a small number of employees with FMS in 2017.

**Table 4:** Binary logistic regression analysis of predictors of PHI purchasing.

Variables	Model 1: Univariate Analysis		Model 2: Multivariable Analysis	
	OR	95% CI	OR	95% CI
Gender (ref = male)				
Female	0.940 **	(0.890, 0.994)		
Age (ref = 18–24)				
25–34	1.369 ***	(1.225, 1.530)	1.193 *	(0.994, 1.432)
35–44	1.828 ***	(1.642, 2.035)	1.599 ***	(1.319, 1.938)
45–54	1.155 ***	(1.037, 1.287)	1.296 ***	(1.063, 1.580)
55–64	0.638 ***	(0.566, 0.720)	0.839	(0.669, 1.052)
≥65	0.236 ***	(0.204, 0.0274)	0.567 ***	(0.407, 0.789)
Education (ref = junior high school and below)				
High school or secondary	2.434 ***	(2.264, 2.619)	1.402 ***	(1.263, 1.557)
University or college	4.295 ***	(4.022, 4.586)	1.813 ***	(1.616, 2.034)
Master degree or above	7.475 ***	(6.363, 8.782)	2.529 ***	(2.023, 3.161)
Marital status (ref = unmarried)				
Married	1.544 ***	(1.076, 1.239)	1.180 ***	(1.047, 1.330)
Household size	0.882 ***	(0.867, 0.898)	0.891 ***	(0.866, 0.916)
Household income (ref = below 50 thousand)				
50–100 thousand	2.005 ***	(1.846, 2.176)	1.314 ***	(1.175, 1.468)
100–150 thousand	3.049 ***	(2.786, 3.337)	1.619 ***	(1.429, 1.834)
150–200 thousand	4.081 ***	(3.674, 4.533)	1.974 ***	(1.709, 2.280)
≥200 thousand	7.357 ***	(6.772, 7.993)	2.856 ***	(2.523, 3.233)
Employment status (ref = not currently working)				
Currently working	1.273 ***	(1.185, 1.367)		
Retired	0.385 ***	(0.346, 0.428)		
Employer type (ref = government or public institution)				
State-owned or collective enterprise	1.299 ***	(1.153, 1.462)	1.470 ***	(1.284, 1.681)
Private or foreign-owned enterprise	0.878 ***	(0.796, 0.968)	1.460 ***	(1.293, 1.647)
Land contracting operator	0.183 ***	(0.157, 0.213)	0.904	(0.747, 1.095)
Other	0.474 ***	(0.402, 0.559)	1.173	(0.970, 1.417)
Hukou (ref = agricultural)				
Non-agricultural	2.348 ***	(2.211, 2.494)		
Unity resident Hukou	2.360 ***	(2.164, 2.574)		
Social medical insurance status (Ref = UEBMI)				
URBMI	0.743 ***	(0.683, 0.808)	1.108	(0.978, 1.255)
NCMS	0.301 ***	(0.281, 0.323)	0.839 ***	(0.745, 0.944)
URRBMI	0.743 ***	(0.632, 0.872)	1.361 ***	(1.092, 1.698)
FMS	0.609 ***	(0.480, 0.773)	0.933	(0.672, 1.296)
Non-SBMI	0.960	(0.877, 1.051)	1.670 ***	(1.458, 1.913)
Other private insurance (ref = no)				
Yes	15.731 ***	(14.731, 16.798)	10.222 ***	(9.395, 11.122)
Geographic region (ref = East)				
Central	0.617 ***	(0.576, 0.662)	0.900 **	(0.821, 0.987)
West	0.602 ***	(0.560, 0.647)	0.818 ***	(0.743, 0.901)
Living area (ref = urban)				
Rural	0.347 ***	(0.322, 0.373)	0.890 **	(0.797, 0.994)
Household medical debt (ref = no)				
Yes	0.296 ***	(0.241, 0.363)	0.718 **	(0.548, 0.942)
Household medical expenses (ref = below 2000 RMB)				
2000–4999	1.045	(0.971, 1.125)	1.129 **	(1.027, 1.242)
5000–9999	0.973	(0.891, 1.062)	1.164 ***	(1.037, 1.306)
10,000–19,999	0.869 ***	(0.787, 0.959)	1.036	(0.908, 1.182)
≥20,000	0.875 ***	(0.798, 0.960)	0.987	(0.863, 1.128)
Health status (ref = good)				
Fair	0.652 ***	(0.612, 0.695)	0.983	(0.902, 1.070)
Poor	0.264 ***	(0.236, 0.295)	0.824 **	(0.694, 0.978)
Constant			0.024 ***	(0.018, 0.031)
Pseudo R^2^			0.204	

Model 2 includes all significant variables through backward stepwise logistic analysis. The number of observations in Model 2 was 58,625. The variance inflation factor (VIF) for all the independent variables in Model 2 ranged from 1.02 to 4.08. OR refers to odds ratio; 95% CI refers to 95% confidence intervals. *** *p* < 0.01, ** *p* < 0.05, * *p* < 0.10

## Appendix A

**Table A1:** Binary logistic regression analysis of predictors of PHI purchasing.

Variables	Model 3: Multivariable Analysis		Model 4: Multivariable Analysis	
	OR	95% CI	OR	95% CI
Gender (ref = male)				
Female	1.014	(0.952, 1.079)	1.020	(0.948, 1.098)
Age (ref = 18–24)				
25–34	1.146 *	(0.993, 1.321)	1.202 **	(1.000, 1.443)
35–44	1.516 ***	(1.301, 1.766)	1.610 ***	(1.325, 1.954)
45–54	1.149 *	(0.983, 1.343)	1.305 ***	(1.069, 1.593)
55–64	0.764 ***	(0.638, 0.914)	0.841	(0.669, 1.057)
≥65	0.388 ***	(0.310, 0.486)	0.569 ***	(0.408, 0.793)
Education (ref = junior high school and below)				
High school or secondary	1.405 ***	(1.289, 1.532)	1.398 ***	(1.258, 1.555)
University or college	1.671 ***	(1.517, 1.841)	1.811 ***	(1.610, 2.038)
Master degree or above	2.067 ***	(1.687, 2.532)	2.538 ***	(2.024, 3.183)
Marital status (ref = unmarried)				
Married	1.200 ***	((1.084, 1.327)	1.180 ***	(1.047, 1.331)
Household size	0.887 ***	(0.866, 0.908)	0.890 ***	(0.866, 0.916)
Household income (ref = below 50 thousand)				
50–100 thousand	1.378 ***	(1.259, 1.509)	1.310 ***	(1.172, 1.465)
100–150 thousand	1.698 ***	(1.530, 1.884)	1.614 ***	(1.425, 1.829)
150–200 thousand	2.013 ***	(1.780, 2.278)	1.977 ***	(1.711, 2.285)
≥200 thousand	3.059 ***	(2.757, 3.394)	2.860 ***	(2.525, 3.239)
Employment status (ref = not currently working)				
Currently working	1.217 ***	(1.118, 1.325)	0.996	(0.817, 1.214)
Retired	0.964	(0.817, 1.137)	1.425	(0.551, 3.688)
Employer type (ref = government or public institution)				
State-owned or collective enterprise			1.472 ***	(1.285, 1.686)
Private or foreign-owned enterprise			1.461 ***	(1.293, 1.652)
Land contracting operator			0.904	(0.746, 1.097)
Other			1.184 *	(0.979, 1.432)
Hukou (ref = agricultural)				
Non-agricultural	0.990	(0.898, 1.090)	0.997	(0.892, 1.115)
Unity resident Hukou	0.968	(0.860, 1.089)	0.941	(0.818, 1.083)
Social Basic Medical Insurance status (ref = UEBMI)				
URBMI	1.092 *	(0.989, 1.204)	1.107	(0.976, 1.255)
NCMS	0.816 ***	(0.730, 0.912)	0.832 ***	(0.730, 0.948)
URRBMI	1.271 ***	(1.062, 1.521)	1.372 ***	(1.099, 1.712)
FMS	0.743 **	(0.567, 0.974)	0.967	(0.695, 1.345)
Non-SBMI	1.750 ***	(1.566, 1.956)	1.664***	(1.450, 1.909)
Other private insurance (ref = no)				
Yes	10.837 ***	(10.091, 11.638)	10.209 ***	(9.382, 11.109)
Geographic region (ref = East)				
Central	0.872 ***	(0.806, 0.944)	0.900 **	(0.820, 0.988)
West	0.855 ***	(0.788, 0.927)	0.819 ***	(0.743, 0.903)
Living area (ref = urban)				
Rural	0.812 ***	(0.738, 0.893)	0.887 **	(0.792, 0.993)
Household medical debt (ref = no)				
Yes	0.704 ***	(0.566, 0.875)	0.708 **	(0.539, 0.931)
Household medical expenses (ref = below 2000 RMB)				
2000–4999	1.070	(0.985, 1.162)	1.127 **	(1.025, 1.240)
5000–9999	1.168 ***	(1.059, 1.288)	1.167 ***	(1.040, 1.310)
10,000–19,999	1.027	(0.920, 1.147)	1.038	(0.908, 1.184)
≥20,000	1.067	(0.961, 1.187)	0.994	(0.870, 1.136)
Health status (ref = good)				
Fair	0.971	(0.903, 1.044)	0.983	(0.903, 1.071)
Poor	0.751 ***	(0.660, 0.854)	0.817 **	(0.689, 0.971)
Constant	0.028 ***	(0.023, 0.034)	0.024 ***	(0.017, 0.033)
Pseudo R^2^	0.210		0.204	

Model 3 includes all the independent variables except employer type. The number of observations in Model 3 was 101,360. The VIF for all the independent variables in Model 3 ranged from 1.05 to 5.86, with all values below the conventional threshold value, and the maximum VIF was below 10. Model 4 includes all the independent variables. The number of observations in Model 4 was 58,479. The VIF for all the independent variables in Model 4 ranged from 1.03 to 5.06, with all values below the conventional threshold value, and the maximum VIF was below 10. OR refers to odds ratio; 95% CI refers to 95% confidence intervals. *** *p* < 0.01, ** *p* < 0.05, * *p* < 0.10

**Table A2:** Binary logistic regression analysis of each set of predictors of PHI purchasing.

Variables	Model 5	Model 6	Model 7
	OR	OR	OR
Gender (ref = male)			
Female	1.001		
Age (ref = 18–24)			
25–34	1.244 ***		
35–44	1.937 ***		
45–54	1.416 ***		
55–64	0.785 ***		
≥65	0.313 ***		
Education (ref = junior high school and below)			
High school or secondary	2.151 ***		
University or college	3.496 ***		
Master degree or above	5.124 ***		
Marital status (ref = unmarried)			
Married	1.397 ***		
Household size	0.856 ***		
Household income (ref = below 50 thousand)			
50–100 thousand		1.350 ***	
100–150 thousand		1.731 ***	
150–200 thousand		2.177 ***	
≥200 thousand		3.344 ***	
Employment status (ref = not currently working)			
Currently working		0.933	
Retired		0.870	
Employer type (ref = government or public institution)			
State-owned or collective enterprise		1.355 ***	
Private or foreign-owned enterprise		1.300 ***	
Land contracting operator		0.650 ***	
Other		0.981	
Hukou (ref = agricultural)			
Non-agricultural		1.162 ***	
Unity resident Hukou		1.094	
Social Basic Medical Insurance status (ref = UEBMI)			
URBMI		0.931	
NCMS		0.639 ***	
URRBMI		1.109	
FMS		1.008	
Non-SBMI		1.389 ***	
Other private insurance (ref = no)			
Yes		11.012 ***	
Geographic region (ref = East)			
Central		0.890 **	
West		0.821 ***	
Living area (ref = urban)			
Rural		0.798 ***	
Household medical debt (ref = no)			
Yes		0.678 ***	
Household medical expenses (ref = below 2000 RMB)			
2000–4999		1.095 *	
5000–9999		1.118 *	
10,000–19,999		0.975	
≥20,000		0.899	
Health status (ref = good)			
Fair			0.652 ***
Poor			0.364 ***
Constant	0.039 ***	0.037 ***	0.071 ***
Pseudo R^2^	0.084	0.191	0.019

Model 5 includes all the predisposing variables. The number of observations in Model 5 was 104,787. The VIF for all the independent variables in Model 5 ranged from 1.00 to 3.98, with all values below the conventional threshold value, and the maximum VIF was below 10. Model 6 includes all the enabling variables. The number of observations in Model 6 was 58,651. The VIF for all the independent variables in Model 6 ranged from 1.02 to 3.80, with all values below the conventional threshold value, and the maximum VIF was below 10. Model 7 includes all the needs-based variables. The number of observations in Model 7 was 105,172. OR refers to odds ratio. *** *p* < 0.01, ** *p* < 0.05, * *p* < 0.10

**Table A3:** Statistical results for model fit of each univariate logistic regression.

Variables	*p*-Value	Chi2	Prob > chi2
Gender (ref = male)		4.80	0.029
Female	0.029		
Age (ref = 18–24)		1692.27	0.000
25–34	0.000		
35–44	0.000		
45–54	0.009		
55–64	0.000		
≥65	0.000		
Education (ref = junior high school and below)		2159.26	0.000
High school or secondary	0.000		
University or college	0.000		
Master degree or above	0.000		
Marital status (ref = unmarried)		16.43	0.000
Married	0.000		
Household size	0.000	202.59	0.000
Household income (ref = below 50 thousand)		2443.90	0.000
50–100 thousand	0.000		
100–150 thousand	0.000		
150–200 thousand	0.000		
≥200 thousand	0.000		
Employment status (ref = not currently working)		844.82	0.000
Currently working	0.000		
Retired	0.000		
Employer type (ref = government or public institution)		1128.21	0.000
State-owned or collective enterprise	0.000		
Private or foreign-owned enterprise	0.009		
Land contracting operator	0.000		
Other	0.000		
Hukou (ref = agricultural)		898.69	0.000
Non-agricultural	0.000		
Unity resident Hukou	0.000		
Social Basic Medical Insurance status (ref = UEBMI)		1420.91	0.000
URBMI	0.000		
NCMS	0.000		
URRBMI	0.000		
FMS	0.000		
Non-SBMI	0.376		
Other private insurance (ref = no)		5399.15	0.000
Yes	0.000		
Geographic region (ref = East)		304.43	0.000
Central	0.000		
West	0.000		
Living area (ref = urban)		967.29	0.000
Rural	0.000		
Health status (ref = good)		804.47	0.000
Fair	0.000		
Poor	0.000		
Household medical debt (ref = no)		203.16	0.000
Yes	0.000		
Household medical expenses (ref = below 2000 RMB)		19.49	0.001
2000–4999	0.000		
5000–9999	0.000		
10,000–19,999	0.000		
≥20,000	0.000		
